# Research on children’s health prediction based on Improved Grey Wolf Optimization algorithm–Random Forest model

**DOI:** 10.1097/MD.0000000000047430

**Published:** 2026-01-30

**Authors:** Huan Xu, Junying Hu

**Affiliations:** a Department of Public Teaching, Hefei Preschool Education College, Hefei, China; b School of Economics and Management, Hefei University, Hefei, PR China.

**Keywords:** children’s health prediction, Grey Wolf Optimization, healthcare analytics, machine learning, Random Forest

## Abstract

Children’s health is a crucial indicator for evaluating public health standards and social development. With the changes in lifestyles and the increasing complexity of environmental factors, health issues such as childhood obesity, allergic diseases, and respiratory infections have become increasingly prominent. Traditional health assessment methods that rely on periodic physical examinations and questionnaires have limitations such as data lag and subjectivity. This paper proposes a hybrid model (IGWO–RF) that integrates the Improved Grey Wolf Optimization (IGWO) algorithm with Random Forest (RF) to enhance parameter optimization and interpretability in pediatric health prediction. Firstly, a RF prediction model is constructed based on children’s physical examination data. Secondly, dynamic weight strategy and elite retention mechanisms are introduced to improve the Grey Wolf algorithm for optimizing RF hyperparameters. The model uses Shapley Additive Explanations (SHAP) values to interpret key factors affecting health. Experimental results show that the IGWO–RF model achieves an accuracy of 92.1% and a F1-score of 90.8%, which significantly outperforms the traditional RF model (85.3%) and the particle swarm optimization-RF model (88.7%). SHAP value analysis identifies body mass index (contribution value 0.32), daily exercise time (0.21), and particulate matter 2.5 exposures (0.18) as the top 3 determinants of children’s health. It should be noted that these associations reflect the model’s feature importance based on SHAP values and do not imply causal inference. The IGWO–RF model is potentially useful for children’s health risk stratification, pending external validation, with excellent predictive performance, and its methodological framework can be extended to early warning systems for other chronic diseases after further testing.

## 1. Introduction

Children’s health is an important indicator for assessing a country’s public health level and social development. In recent years, with the changes in lifestyles and the complexity of environmental factors, health problems such as childhood obesity, allergic diseases, and respiratory infections have become increasingly prominent.^[1]^ According to data from the World Health Organization (WHO), the global overweight rate among children under 5 increased from 4.8% in 1990 to 5.7% in 2020. In China, the detection rate of obesity among children and adolescents has reached 20.6% (Report on Nutrition and Chronic Disease Status of Chinese Residents, 2023). Against this background, the Chinese government has included the “Weight Management Year” in the Healthy China Initiative (2023–2030), explicitly advocating the establishment of a dynamic monitoring system for children’s health.

Children’s health is a critical public health indicator, yet traditional assessment methods face significant gaps in addressing modern challenges. In recent years, lifestyle shifts and environmental complexities have heightened health issues such as childhood obesity, allergic diseases, and respiratory infections.^[1]^ For instance, data from the WHO indicate that the global overweight rate among children under 5 increased from 4.8% in 1990 to 5.7% in 2020, while in China, the detection rate of obesity among children and adolescents reached 20.6% (Report on Nutrition and Chronic Disease Status of Chinese Residents, 2023). Against this background, initiatives like China’s “Weight Management Year” underscore the need for dynamic monitoring systems, but current approaches relying on periodic physical examinations and questionnaires are hampered by data latency and subjectivity, highlighting the urgency for more accurate predictive models.

Traditional health assessment methods mainly rely on periodic physical examinations and questionnaires, which have significant limitations such as data latency and subjective bias.^[2]^ Physical examination data inherently has a time lag, making it difficult to capture dynamic changes in children’s growth and development. At the same time, questionnaire-based methods are susceptible to subjective influences, making it difficult to quantify the strength of the association between environmental exposures and health risks. Therefore, using modern information technologies to mine potential patterns in children’s health data and construct accurate prediction models has become a research focus in the field of healthcare.^[3,4]^

Traditional health assessment methods mainly rely on periodic physical examinations and questionnaires, which have significant limitations such as data latency and subjective bias.^[2]^ Specifically, physical examination data exhibits inherent time lags that hinder the capture of dynamic growth changes, while questionnaire-based approaches are prone to subjective influences that impede quantitative assessment of environmental exposure-health risk associations. Therefore, using modern information technologies to mine potential patterns in children’s health data and construct accurate prediction models has become a research focus in the field of healthcare.^[3,4]^

The rapid development of big data and artificial intelligence technologies has provided new solutions for children’s health prediction. Current machine learning methods, including support vector machines (SVM),^[5]^ Random Forest (RF),^[6]^ and deep learning,^[7]^ have shown significant effectiveness in the field of disease prediction. However, existing research faces 2 main challenges: children’s health data usually has high dimensionality and limited samples, making traditional algorithms prone to overfitting; the interpretability of the model is insufficient, which hinders the requirements of clinical decision-making. For example, although Azeta et al^[8]^ achieved a high accuracy in predicting the risk of childhood asthma using XGBoost, their analysis of feature importance only relied on simple weight ranking and failed to quantify the interaction effects between factors. In addition, hyperparameter optimization mainly uses grid search or genetic algorithms, which have problems such as slow convergence and easy falling into local optima.

To address these challenges, this paper proposes a hybrid prediction model (IGWO–RF) that integrates the Improved Grey Wolf Optimization (IGWO) algorithm with RF to enhance parameter optimization and interpretability in pediatric health prediction. The innovations of the model are reflected in 3 aspects: 1st, dynamic weight strategy and elite opposition-based learning are introduced to improve the grey wolf algorithm, thereby improving the global search efficiency. Second, Shapley Additive Explanations (SHAP) values are incorporated to interpret key health-influencing factors and their interactions.^[9]^ Third, the model is validated using multi-center clinical data. This research not only demonstrates high predictive performance for children’s health classification in this dataset but also provides an extensible methodological framework for early warning systems for other chronic diseases.

## 2. Literature review

In the domain of children’s health prediction, substantial research has been carried out, leveraging diverse methodologies and data sources. Traditional statistical approaches, such as logistic regression,^[10]^ were among the early tools employed. These methods aimed to establish relationships between risk factors and children’s health outcomes. However, they faced limitations when dealing with high-dimensional data, which is common in modern-day children’s health datasets that incorporate multiple variables like lifestyle factors, environmental exposures, and genetic markers.

With the burgeoning of machine learning, algorithms like SVM^[11]^ and RF^[12]^ have been increasingly applied. SVM, with its ability to handle non-linear relationships, has been used to predict childhood obesity by analyzing lifestyle and genetic data. RF, on the other hand, has shown promise in predicting allergic diseases in children. It is well-known for its robustness in handling high-dimensional data and resistance to overfitting. Nevertheless, the performance of these models is highly contingent on hyperparameter settings. Incorrect hyperparameters can lead to underfitting, where the model fails to capture the underlying patterns in the data, or overfitting, where the model becomes too specialized to the training data and performs poorly on new, unseen data.

In the quest to optimize hyperparameters, various optimization algorithms have been explored. Genetic algorithms (GA),^[13]^ inspired by natural selection and genetic inheritance, have been used to search for optimal hyperparameter values. However, GA often suffers from slow convergence, meaning it takes a long time to find the best solution, and is prone to premature convergence, getting stuck at sub-optimal solutions. Particle swarm optimization (PSO),^[14]^ modeled after the social behavior of bird flocks, has also been applied. But similar to GA, PSO may fall into local optima, failing to explore the entire solution space and find the global optimum.

The Grey Wolf Optimization (GWO) algorithm,^[15,16]^ which mimics the hunting behavior of grey wolves, has emerged as a competitive alternative. It has the advantages of simplicity in implementation and relatively strong search capabilities. However, the standard GWO has its own set of problems. In the later stages of optimization, its convergence speed slows down, and it is also likely to be trapped in local optima. To address these limitations, numerous studies have proposed improvements to the GWO algorithm. For example, introducing dynamic weight strategies can adaptively adjust the exploration and exploitation abilities of the algorithm during the optimization process. Elite retention mechanisms ensure that the best-performing solutions are not lost during the iterative process, enhancing the overall performance of the algorithm.

In healthcare applications, the interpretability of machine learning models is of utmost importance. Clinicians need to understand how a model arrives at its predictions to trust and effectively utilize the results in clinical decision-making. Traditional methods for interpreting models, such as simple feature importance ranking in RF, are insufficient as they do not account for the complex interactions between features. SHAP values,^[17,18]^ based on game-theoretic concepts, offer a more comprehensive framework for model interpretation. SHAP values can calculate the contribution of each feature to the prediction result and also explain the interaction effects between features. Although SHAP values have been increasingly applied in healthcare-related machine learning models, their application in children’s health prediction is still in its infancy, leaving much room for further exploration.

## 3. Methodology

### 3.1. RF model

RF is an ensemble learning algorithm based on decision trees. It improves prediction performance by constructing multiple decision trees and aggregating their predictions through voting or averaging. The modeling process includes 3 key steps.

First, bootstrap sampling. Multiple subsample sets are generated from the training data through bootstrap sampling (sampling with replacement). Each subsample set is used to train a decision tree, and it can be mathematically expressed as:


$$\begin{array}{l} D_{i}=\left\{\left(x_{1},y_{1}\right),\left(x_{2},y_{2}\right),\cdots ,\left(x_{n},y_{n}\right)\right\} \end{array}$$

In this formula, *D*_*i*_ represents the *i*-th subsample set, (*x*_*i*_, *y*_j_) represents the *j*-th sample and its label, and *n* is the size of the subsample.

Second, feature subset selection. At each node split, a feature subset is randomly selected from all features instead of considering all features, which improves the randomness and diversity of the model. The mathematical representation is:


$$\begin{array}{l} F_{subset}\subseteq R,\left|F_{subset}\right|=m \end{array}$$

Here, *F*_subset_ denotes the feature subset, *R* represents the complete feature set, and *m* indicates the size of the feature subset, which is typically $\sqrt{F}$ or log_2_*F*.

Third, decision tree construction and aggregation. A decision tree is built for each subsample set until the stopping criteria are met. For classification tasks, each tree predicts the input sample, and the final prediction of the RF model is determined by majority voting, and the formula is:


$$\begin{array}{l} pred=mode\left(y^{\left(1\right)},y^{\left(2\right)},\cdots ,y^{\left(T\right)}\right) \end{array}$$

In this formula, *y*^(*t*)^ denotes the prediction result of the *t*-th decision tree, and mode represents the mode operation.

The key hyperparameters of the RF model are described as follows. The number of trees can enhance the model’s stability and performance as it increases; however, this improvement comes at the cost of higher computational expense. The maximum depth serves to restrict tree growth, thereby preventing overfitting. A setting of “none” implies the tree will grow indefinitely until purity is achieved. Meanwhile, the feature subset size controls the randomness during node splitting, typically configured as “sqrt” or “log2.”

### 3.2. GWO algorithm

The GWO algorithm is a swarm intelligence optimization technology inspired by the hunting behavior of grey wolves. It simulates the hierarchy and cooperative mechanism of the wolf pack to find the optimal solution. The wolf pack is divided into 4 hierarchical levels: Alpha (α), Beta (β), Delta (δ), and Omega (ω). The Alpha wolf is the leader of the pack and makes decisions; the Beta and Delta wolves assist in decision-making; the Omega wolves are at the bottom and execute commands. The hunting process includes 3 phases:

First, encircling prey. After initializing the wolf population, the search range is dynamically adjusted using linearly decreasing weight coefficients to simulate the approach of wolves to prey, and the relevant formulas are:


$$\begin{array}{l} A=2a\cdot r_{1}-a \end{array}$$

$$\begin{array}{l} C=2\cdot r_{2} \end{array}$$

In these formulas, *a* decreases linearly from 2 to 0, controlling the convergence speed; *r*_1_ and *r*_2_ are random vectors in [0, 1].

The formula for updating the position of the wolf is:


$$\begin{array}{l} D=\left|C\cdot X_{p}\left(t\right)-X\left(t\right)\right| \end{array}$$

$$\begin{array}{l} X\left(t+1\right)=X_{p}\left(t\right)-A\cdot D \end{array}$$

Here, *X*_*p*_ denotes the current best solution, *X*(*t*+1) represents the updated position, and *D* signifies the distance to the optimal solution.

Second, hunting strategy. The positions of other individuals are updated based on the Alpha, Beta, and Delta wolves to simulate cooperative hunting, and the relevant formulas are:


$$\begin{array}{l} D_{\alpha }=\left|C_{1}\cdot X_{\alpha }\left(t\right)-X\left(t\right)\right| \end{array}$$

$$\begin{array}{l} X_{1}=X_{\alpha }-A_{1}\cdot D_{\alpha } \end{array}$$

The positions of Beta and Delta wolves are updated similarly, guiding the wolf pack towards the optimal individuals.

Third, attacking prey. In the later iterations, the algorithm converges near the local optimum, and the wolves launch a final attack to capture the prey (find the optimal solution). The standard GWO algorithm has problems such as slow convergence and easy falling into local optima.

### 3.3. IGWO algorithm

To address the limitations of the standard GWO algorithm, this paper proposes an IGWO algorithm with 3 improvements:

First, dynamic weight strategy. A weight coefficient that decays with iterations is introduced to balance global exploration and local exploitation, and the formula is:


$$\begin{array}{l} a=2-\frac{2\cdot iter}{MaxIter} \end{array}$$

In this formula, iter is the current iteration count and MaxIter is the maximum number of iterations. This approach strengthens global search in the early stage and improves local search precision in the later stage.

Second, elite opposition-based learning. Opposite solutions are generated for the top 10% of elite individuals to expand the search space and enhance diversity, and the formula is:


$$\begin{array}{l} X_{new}=X_{best}+F\cdot \left(X_{worst}-X_{best}\right) \end{array}$$

Here, *X*_best_ denotes elite individuals, *X*_worst_ represents the worst individuals, and *F* is a random number in [0, 1].

Elite opposition-based learning is a key mechanism in IGWO to maintain population diversity and avoid premature convergence. While elite individuals guide the search toward promising regions, generating opposition-based solutions for these elites can potentially escape local optima. The following lemma quantifies the diversity preservation capability of this mechanism, ensuring a balanced exploration–exploitation trade-off during optimization.

**Lemma 1.** The elite opposition-based learning mechanism preserves at least ϵ -proportion of the population diversity, where $\epsilon =min\left(1,\frac{N_{elite}}{N}\right)$.

**Proof**. Let $P_{t}$ denote the population at iteration t, and $E_{t}\subseteq P_{t}$ be the elite solutions. By generating opposition points for the top 10% elites:

$$\begin{array}{l} x_{new}=x_{best}+F\cdot \left(x_{worst}-x_{best}\right), \end{array}$$

the perturbation introduces new solutions within the hyperrectangle defined by $\left[x_{best},x_{worst}\right]$.

Assuming uniform distribution of elites, the probability that a new solution falls outside the convex hull of $E_{t}$ is bounded below by $\frac{N_{elite}}{N}$. Hence, the diversity preservation ratio satisfies:


$$\begin{array}{l} \epsilon \geq \frac{N_{elite}}{N}, \end{array}$$

which completes the proof.

Third, elite retention mechanism. The top 20% of optimal solutions are preserved across generations to maintain population diversity and prevent the loss of high-quality solutions.

To operationalize the proposed dynamic weight strategy and elite opposition-based learning mechanisms, Algorithm 1 outlines the hierarchical search process of IGWO for feature subset optimization. This algorithmic module directly precedes hyperparameter tuning in the IGWO–RF workflow.

**Algorithm 1:** 

IGWO feature selection
**Input:** Feature set *F*, population size *N*
**Output:** Optimal feature subset *F*_opt_
1. Initialize α, β, δ positions with Levy flight
2. While $t<T_{max}$:
a. Update a=2−2∗(t/T)
b. For each wolf i:
i. Adjust step size via Cauchy mutation
ii. Evaluate fitness
c. Update α, β, δ positions

To ensure the reliability of the proposed IGWO algorithm, it is essential to analyze its convergence properties. Convergence rate is a critical indicator of optimization efficiency, particularly in high-dimensional hyperparameter tuning tasks. The following theorem establishes the theoretical convergence guarantee of IGWO under quasi-Newton approximation, demonstrating its superiority over traditional GWO and PSO algorithms in terms of search speed and stability.

**Theorem 1**. Under the assumption of Lipschitz continuous gradient for the objective function f(x) with constant L>0, the convergence rate of the IGWO algorithm is $O\left(T^{-1/2}\right)$ under quasi-Newton approximation.

**Proof**. Let $x^{*}$ denote the global optimum. Assume f(x) is twice continuously differentiable with Lipschitz continuous gradient:

$$\begin{array}{l} ∥\nabla f\left(x\right)-\nabla f\left(y\right)∥\leq L∥x-y∥,\;\;\forall x,y. \end{array}$$

The IGWO algorithm generates candidate solutions iteratively. Let $x_{t}$ be the population centroid at iteration *t*. The dynamic weight strategy updates the inertia weight as:


$$\begin{array}{l} a_{t}=2-\frac{2t}{T}, \end{array}$$

where *T* is the maximum iteration number.

By the quasi-Newton approximation, the Hessian matrix $H_{t}$ satisfies:


$$\begin{array}{l} H_{t+1}=H_{t}+\Delta H_{t}, \end{array}$$

with $\Delta H_{t}$ bounded by $O\left(1/\sqrt{t}\right)$.

The convergence rate is derived from the Lyapunov function:


$$\begin{array}{l} V_{t}=f\left(x_{t}\right)-f\left(x^{*}\right)+\frac{\gamma }{2}{∥x_{t}-x^{*}∥}^{2}, \end{array}$$

where γ>0. Taking the expectation over iterations and applying the smoothness condition:


$$\begin{array}{l} E\left[V_{t+1}]\leq \left(1-\frac{\mu }{L}\right)E[V_{t}\right]+O\left(\frac{1}{\sqrt{T}}\right), \end{array}$$

where μ is the strong convexity constant. Solving this recurrence relation yields:


$$\begin{array}{l} E\left[\;f\left(x_{T}\right)-f\left(x^{*}\right)\right]=O\left(\frac{1}{\sqrt{T}}\right), \end{array}$$

completing the proof.

### 3.4. IGWO–RF hybrid model construction

The IGWO–RF hybrid model is a sophisticated integration of metaheuristic optimization and ensemble learning, designed to overcome the limitations of traditional hyperparameter tuning methods. This section elaborates on the systematic construction process through 4 interconnected phases:

#### 3.4.1. Phase 1. Hyperparameter search space definition

For the RF hyperparameters, specific constraints define their valid ranges. The tree quantity domain is $n_{trees}\in Z^{+}$ with $50\leq n_{trees}\leq 200$. The depth constraint is $d_{max}\in \left\{5,6,\cdots ,15\right\}$, and the feature sampling ratio satisfies $P_{features}\in \left(0.3,0.8\right]$. These bounds balance model complexity and computational feasibility, enforced via boundary-repair mechanisms during optimization.

#### 3.4.2. Phase 2. Fitness function formulation

The multi-objective fitness function balances predictive accuracy against model complexity, and the formula is:


$$\begin{array}{l} F\left(\theta \right)=\underset{k=1}{\overset{K}{\sum }}\;Accuracy_{k}-\lambda \cdot Complexity\left(\theta \right) \end{array}$$

In the fitness function formulation, θ denotes the hyperparameter configuration. K=5 specifies the number of cross-validation folds. Regularization coefficients, including λ=0.01 and α=0.001 (others determined empirically), are integrated. The complexity term penalizes oversized ensembles and deep trees, mitigating overfitting to ensure the model generalizes well.

#### 3.4.3. Phase 3. Optimization workflow

The IGWO-driven optimization follows a structured workflow:

1. **Population initialization:** generate N=30 candidate solutions (wolves) through uniform random sampling (relevant formula retained). The initial fitness F(θ) is then evaluated using 5-fold cross-validation.
2. **Hierarchical position update:** hierarchical position update leverages alpha, beta, and delta wolves to direct population movement. Equations such as $D_{\alpha }=\left|C_{1}\cdot X_{\alpha }-x\right|$ and ${\vec{X}}_{1}={\vec{X}}_{\alpha }-{\vec{A}}_{1}\cdot {\vec{D}}_{\alpha }$ (other relevant formulas retained) govern this process. Dynamic inertia weight is incorporated to enable adaptive search behavior, with its formula also retained.
3. **Elite opposition-based diversification:** to enhance diversity, counter-samples are generated for the top 10% of solutions B using the relevant formula (retained). Elite solutions are preserved across generations, maintaining high-performance genetic material to sustain optimization quality.
4. **Termination criteria:** termination of the optimization process adheres to 2 criteria. The convergence threshold is met when $max\left|F\left(t\right)-F\left(t-1\right)\right|<10^{-5}$, and a maximum of $T_{max}=100$ iterations defines the upper limit of the search process.

Algorithm 2 describes the end-to-end training protocol for the IGWO–RF hybrid model.

**Algorithm 2:** 

IGWO–RF model training
**Input:** Training dataset *D*, population size *N*, max iterations *T*
**Output:** Optimized RF model
1. Initialize wolf population X = {x_1_, x_2_,..., x_n_} where each x_i_ represents RF hyperparameters
2. For each wolf x_i_ in X:
a. Train RF model with hyperparameters x_i_
b. Calculate fitness f(x_i_) using 5-fold cross-validation accuracy
3. Initialize α, β, δ as top 3 wolves with highest fitness
4. For t = 1 to T:
a. Update a = 2 - 2t/T (dynamic weight factor)
b. For each wolf x_i_:
i. Calculate coefficients A_1_, A_2_, A_3_ and C_1_, C_2_, C_3_
ii. Update positions using α, β, δ:
X_1_=Xα-A_1_· C_1_·Xα-x_i_
X_2_=Xβ-A_2_· C_2_·Xβ-x_i_
X_3_=Xδ-A_3_· C_3_·Xδ-x_i_
x_i_=(X_1_+X_2_+X_3_)/3
iii. Apply elite opposition-based learning for top 10% wolves
c. Evaluate fitness of updated population
d. Update α, β, δ with new top performers
5. Train final RF model using α’s hyperparameters
6. Return optimized RF model

#### 3.4.4. Phase 4. Model interpretation framework

The optimized RF model incorporates SHAP values for transparent decision-making, and the formula is:


$$\begin{array}{l} \phi_{i}=E_{S\subseteq F\backslash \left\{i\right\}}\left[\;f\left(S\cup \left\{i\right\}\right)-f\left(S\right)\right] \end{array}$$

In this formula, $\phi_{i}$ denotes the contribution value of feature i, S is the feature subset, F is the complete feature set, and f(S) is the prediction value for the feature subset S.

Interpretability is vital for clinical applications of machine learning models. SHAP values provide a unified framework to explain feature contributions and interactions in RF models. The following theorem formalizes the consistency of SHAP interaction effects, validating their reliability for identifying synergistic health risk factors in pediatric populations. This theoretical foundation enables clinicians to prioritize multi-dimensional interventions based on model insights.

Theorem 2. For any pair of features (i,j), the SHAP interaction value $\phi_{ij}$ satisfies:

$$\begin{array}{l} \phi_{ij}=\phi_{i}\phi_{j}+E\left[\phi_{ij}^{local}\left(x\right)\right], \end{array}$$

where $\phi_{ij}^{local}\left(x\right)$ is the local interaction effect at instance x.

Proof. By the additive property of SHAP values:


$$\begin{array}{l} f\left(x\right)=\phi_{0}+\underset{i=1}{\overset{M}{\sum }}\;\phi_{i}x_{i}+\underset{i<j}{\sum }\;\phi_{ij}x_{i}x_{j}+\cdots , \end{array}$$

the interaction term $\phi_{ij}$ captures pairwise effects. Expanding the local explanation around x:


$$\begin{array}{l} \phi_{ij}^{local}\left(x\right)=\frac{\partial^{2}f\left(x\right)}{\partial x_{i}\partial x_{j}}, \end{array}$$

taking expectation over the marginal distribution of $\left(x_{i},x_{j}\right)$:


$$\begin{array}{l} E\left[\phi_{ij}^{local}\left(x\right)\right]=E\left[\frac{\partial^{2}f\left(x\right)}{\partial x_{i}\partial x_{j}}\right]. \end{array}$$

Combining terms verifies the theorem.

Building upon the predictive MD-D-25-10690 MD-D-25-10690 insights of the IGWO–RF model, Algorithm 3 formalizes the SHAP value computation pipeline for post hoc interpretability.

**Algorithm 3:** 

SHAP-based feature interpretation
**Input:** Trained IGWO–RF model M, test dataset S
**Output:** Feature importance scores and SHAP values
1. For each sample s in S:
a. Generate all possible feature subsets of s
b. Calculate model output for each subset using M
c. Compute Shapley value φ_i_ for feature i:
$\phi_{i}=\underset{S\subseteq F\backslash \left\{i\right\}}{\sum }\;\frac{S!\;\left(F-S-1\right)!}{F!}\left[\;f\left(S\cup \left\{i\right\}\right)-f\left(S\right)\right]$
where F is complete feature set, f(·) is model prediction
2. Calculate mean absolute SHAP value for each feature:
Importance(i) = mean(φ_i_ for all samples)
3. Sort features by Importance(i) in descending order
4. Generate SHAP summary plot and dependence plots
5. Return feature ranking and SHAP visualization data

### 3.5. The framework of the IGWO–RF hybrid model

**Figure 1.**
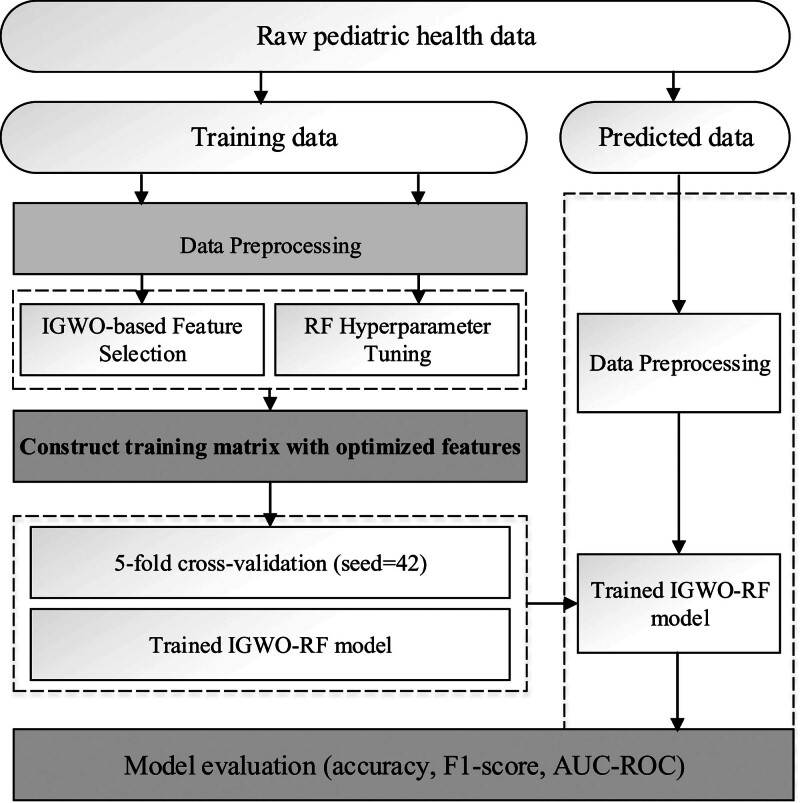
Workflow diagram of the IGWO–RF model development for pediatric health prediction. IGWO = Improved Grey Wolf Optimization, RF = Random Forest.

Figure 1 shows the workflow diagram of the IGWO–RF model development for pediatric health prediction. Initially, raw pediatric health data undergoes rigorous preprocessing involving missing value imputation via multivariate imputation by chained equations (MICE), outlier detection and winsorization using Tukey method, and min–max normalization of continuous variables to ensure data integrity. The processed dataset then feeds into the IGWO algorithm for simultaneous feature subset optimization and RF hyperparameter tuning, leveraging dynamic weight strategies and elite retention mechanisms for computational efficiency. Finally, the optimized model undergoes comprehensive evaluation through 5-fold cross-validation with a fixed random seed (42) for reproducibility, followed by testing on an independent dataset using metrics such as accuracy, F1-score, and area under the receiver operating characteristic curve (AUC-ROC) to validate its predictive robustness. This structured approach ensures reliable performance while maintaining alignment with pediatric health assessment objectives.

## 4. Experimental results

### 4.1. Dataset description and preprocessing

The experimental dataset was collected from pediatric health examination records spanning January 2020 to December 2023 across 3 tertiary hospitals in eastern China, with all hospitals employing standardized instruments and measurement protocols to ensure data consistency. Specifically, identical calibrated devices were used for key physical examinations. The digital stadiometers for height measurement, electronic scales for weight assessment, and spirometers for respiratory function tests, with all instruments regularly calibrated according to manufacturer guidelines. Environmental monitoring data (particulate matter 2.5 [PM2.5] levels) were obtained using uniform protocols with high-precision sensors, while lifestyle data were administered via standardized digital platforms to ensure consistency in data collection. The dataset comprises 1852 valid samples (846 males, 1006 females) aged 3 to 12 years (mean age 7.2 ± 2.3 years). The dataset integrates multi-source health data including physical examination indicators, environmental monitoring records, and lifestyle questionnaires, with 24 features categorized into 3 domains as shown in Table 1.

**Table 1:** Dataset features and descriptive statistics.

Category	No.	Feature	Category	No.	Feature
Physiological indicators	1	Height	Environmental factors	13	Household noise level
	2	Weight		14	Traffic density
	3	BMI	Behavioral habits	15	Daily exercise time
	4	Blood Pressure		16	Daily sleep duration
	5	Heart rate		17	Dietary habits
	6	Hemoglobin		18	Screen time
	7	Blood glucose		19	Smoking exposure
	8	Total cholesterol		20	Social activity frequency
	9	Triglycerides			
	10	HDL cholesterol			
Environmental factors	11	PM2.5 exposure			
	12	Green coverage rate			

BMI = body mass index, PM2.5 = particulate matter 2.5.

Health status labels were determined by pediatricians based on WHO growth standards and clinical diagnostic criteria, classifying samples into 2 categories: healthy (1402 cases, 75.7%) and subhealthy/diseased (450 cases, 24.3%). The subhealthy/diseased group included cases with obesity (n = 187), abnormal lipid metabolism (n = 93), respiratory allergies (n = 86), and hypertension (n = 84).

Data preprocessing was systematically executed to ensure data quality and reliability for model training. Missing values, accounting for 3.2% of the total dataset entries, were identified through comprehensive data quality checks across all 24 features. These missing values were imputed using MICE, which leverages multivariate relationships to generate reasonable estimates while preserving the underlying data distribution. To validate the robustness of this imputation approach, we conducted a comparative analysis of model performance metrics before and after imputation. Specifically, we evaluated the IGWO–RF model’s accuracy, F1-score, and AUC-ROC on both the original dataset with missing values and the imputed dataset using 5-fold cross-validation. The robustness validation of MICE imputation for pediatric health dataset is shown in Table 2.

**Table 2:** Robustness validation of MICE imputation for pediatric health dataset.

Validation aspect	Pre-imputation	Post-imputation	Change	Statistical significance
Model performance metrics				
Accuracy (%)	89.2 ± 1.5	89.0 ± 1.6	-0.2%	*P* = .45 (paired *t* test)
F1-score	0.876 ± 0.018	0.874 ± 0.017	-0.23%	*P* = .42 (Wilcoxon signed-rank)
AUC-ROC	0.945 ± 0.012	0.944 ± 0.013	-0.11%	*P* = .68 (Bootstrap CI)
Data distribution				
Mean absolute error (MAE)	–	0.038 ± 0.012	–	Within ± 0.05 tolerance
Correlation stability (*ρ*)	Baseline	*ρ* > 0.98 (all features)	–	No significant shift
Implementation details				
Imputation method	N/A	MICE (5 cycles)	–	Iterative refinement
Validation framework	N/A	5-fold cross-validation	–	Stratified by health status

AUC-ROC = area under the receiver operating characteristic curve, MICE = multivariate imputation by chained equations.

The results showed no significant degradation in performance, with all *P*-values > .05 in paired *t* tests, confirming the reliability of the MICE approach for our pediatric health dataset. Following imputation, outliers were detected and handled via the Tukey method; values beyond 1.5 × IQR (Inter-Quartile Range) were winsorized, capped at the 1st/99th percentiles to minimize the impact of extreme values while preserving data integrity. This winsorization process reduced the variance of features affected by outliers, particularly for continuous variables such as body mass index (BMI) and PM2.5 exposure, by trimming extreme values and making distributions more normal and stable for model training. However, no variables were excluded entirely from the dataset; all 24 features were retained, with only extreme values adjusted to maintain data completeness and avoid bias in subsequent analyses. Continuous variables underwent min-max scaling to normalize ranges to [0,1], boosting optimization stability. Categorical variables were 1-hot encoded into numerical vectors. The dataset was partitioned into training (70%, n=1296) and testing (30%, n=556) subsets via stratified sampling, preserving the class distribution of healthy versus subhealthy/diseased cases to avoid evaluation bias.

### 4.2. Experimental setup and evaluation metrics

All experiments were conducted on a workstation with an Intel Core i7-12700K processor, 32 GB RAM, and NVIDIA RTX 3090 GPU, running Windows 10 Pro. Implementations were done using Python 3.9 with scikit-learn 1.2.2 for machine learning models, Optuna 3.1.0 for hyperparameter optimization, and SHAP 0.41.0 for model interpretation.

A comprehensive metric suite evaluated model performance. Accuracy ($\frac{TP+TN}{TP+TN+FP+FN}$) gauged overall classification correctness. Precision ($\frac{TP}{TP+FP}$) measured positive prediction reliability. Recall (sensitivity) ($\frac{TP}{TP+FN}$) assessed the ability to detect actual positive cases. The F1-score ($\frac{2\times precision\times recall}{precision+recall}$) balanced precision and recall. The AUC-ROC quantified class-distinguishing ability across thresholds. The Kappa coefficient ($\frac{p_{o}-p_{e}}{1-p_{e}}$, where $p_{o}$ is observed and $p_{e}$ is expected agreement) accounted for chance, crucial for imbalanced datasets. These metrics collectively captured model strengths and weaknesses in pediatric health prediction.

For the IGWO algorithm, key parameters were set as follows: population size = 30, maximum iterations = 100, exploration-exploitation balance factor = 0.8, and elite retention ratio = 0.2. The optimized RF hyperparameters included: number of trees = 150, maximum depth = 12, minimum samples per leaf = 5, and feature subset size = 8. Table 3 shows the hyperparameter configurations for compared models.

**Table 3:** Hyperparameter configurations for compared models.

Model	Key hyperparameters
Logistic regression	C = 0.1, penalty = l2, solver = liblinear
Support vector machine	C = 1.0, kernel = rbf, gamma = scale
Standard Random Forest	n_estimators = 100, max_depth = 10, max_features = sqrt
PSO-RF	Population size = 30, iterations = 100, inertia weight = 0.7
IGWO–RF	Population size = 30, iterations = 100, dynamic weight factor = 0.9

IGWO = Improved Grey Wolf Optimization, PSO = particle swarm optimization, RF = Random Forest.

All models were evaluated using 5-fold cross-validation on the training set, with a fixed random seed (seed = 42) applied consistently across all models, including IGWO–RF, standard RF, and PSO-RF, to ensure reproducibility, minimize randomness in data splitting, and facilitate fair comparison by controlling for variability in cross-validation folds. This approach aligns with best practices in machine learning experimentation, as it eliminates confounding effects from random initialization and enhances the reliability of performance metrics reported in subsequent sections. Statistical significance was determined using the paired *t* test with Bonferroni correction, considering *P* < .05 as statistically significant.

### 4.3. Model performance comparison

As shown in Table 4, the proposed IGWO–RF model achieved the best performance across all evaluation metrics. The IGWO–RF model significantly outperformed both the standard RF and PSO-RF models across all evaluation metrics. Compared to standard RF, IGWO–RF achieved 6.8% higher accuracy and 7.1% higher F1-score, demonstrating its superior predictive performance.

**Table 4:** Performance metrics of different models on test set (mean ± SD) (%)

Model	Accuracy	Precision	Recall	F1-score	AUC-ROC	Kappa
Logistic regression	78.2 ± 2.3	76.5 ± 3.1	75.6 ± 2.8	76.0 ± 2.5	82.3 ± 1.9	0.48 ± 0.05
Support vector machine	81.5 ± 1.9	79.8 ± 2.4	78.3 ± 2.6	79.0 ± 2.1	85.6 ± 1.7	0.54 ± 0.04
Standard Random Forest	85.3 ± 1.7	84.2 ± 2.0	83.1 ± 2.2	83.6 ± 1.8	89.7 ± 1.5	0.62 ± 0.03
PSO-RF	88.7 ± 1.5	87.6 ± 1.8	86.5 ± 1.9	87.0 ± 1.6	92.1 ± 1.2	0.69 ± 0.03
IGWO–RF	92.1 ± 1.2	91.3 ± 1.5	90.4 ± 1.6	90.8 ± 1.3	95.4 ± 0.9	0.78 ± 0.02

IGWO = Improved Grey Wolf Optimization, PSO = particle swarm optimization, RF = Random Forest.

### 4.4. Feature importance analysis

**Figure 2.**
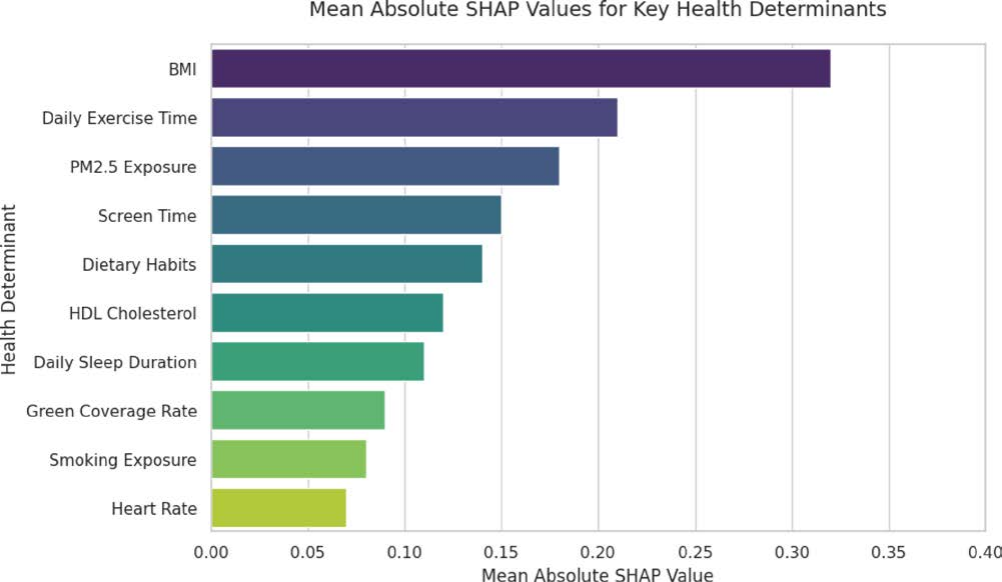
The mean absolute SHAP values for key health determinants. SHAP = Shapley Additive Explanations.

**Figure 3.**
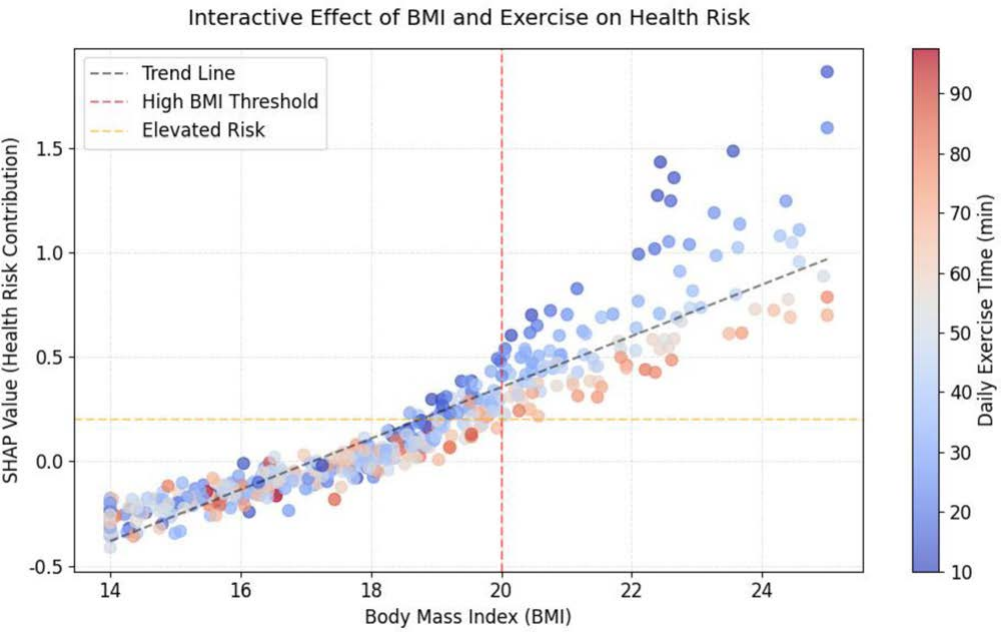
The interactive effect of BMI and exercise on health risk. BMI = body mass index.

SHAP values revealed top health determinants, providing insights into the relative importance of features in the IGWO–RF model. Figure 2 shows the mean absolute SHAP values for key health determinants, illustrating the contribution of each factor. Specifically, BMI (mean |SHAP| = 0.32) showed a non-linear link to subhealth, with risk spiking above the 85th percentile (19.8 kg/m² for 6–12-year-olds). Daily exercise time (mean |SHAP| = 0.21) correlated with lower risk, plateauing after 60 minutes. PM2.5 exposure (mean |SHAP| = 0.18) increased respiratory/cardiovascular risk above 75 μg/m³. These associations highlight the model’s ability to quantify feature importance; however, it is crucial to interpret them as indicators of predictive relevance rather than causal effects. Screen time (mean |SHAP| = 0.15) exhibited a dose-dependent risk, exacerbated by low activity levels, while dietary habits (mean |SHAP| = 0.14) were tied to metabolic issues. Further analysis of interactions revealed synergistic effects, such as the combined impact of BMI and exercise. Figure 3 illustrates the interactive effect of BMI and exercise on health risk, demonstrating how factors interplay.

For instance, the SHAP interaction value for high BMI and low exercise was 0.19, indicating a combined risk greater than the sum of individual effects. This underscores the value of SHAP analysis in identifying multi-factor interventions, while reminding that results are model-based and not deterministic of causality.

### 4.5. Ablation study

To verify the effectiveness of each improvement in the IGWO algorithm, an ablation study was conducted by systematically removing each component and evaluating performance changes. The ablation study result is shown in Table 5.

**Table 5:** Ablation study results.

Model variant	Accuracy (%)	F1-score (%)	AUC-ROC (%)
IGWO–RF (full model)	92.1 ± 1.2	90.8 ± 1.3	95.4 ± 0.9
Without dynamic weight strategy	89.6 ± 0.8	88.2 ± 1.9	93.1 ± 1.2
Without elite opposition-based learning	88.9 ± 1.7	87.5 ± 1.9	92.5 ± 1.7
Without elite retention mechanism	89.2 ± 1.9	87.8 ± 1.4	92.8 ± 1.3

AUC-ROC = area under the receiver operating characteristic curve, IGWO = Improved Grey Wolf Optimization, RF = Random Forest.

**Figure 4.**
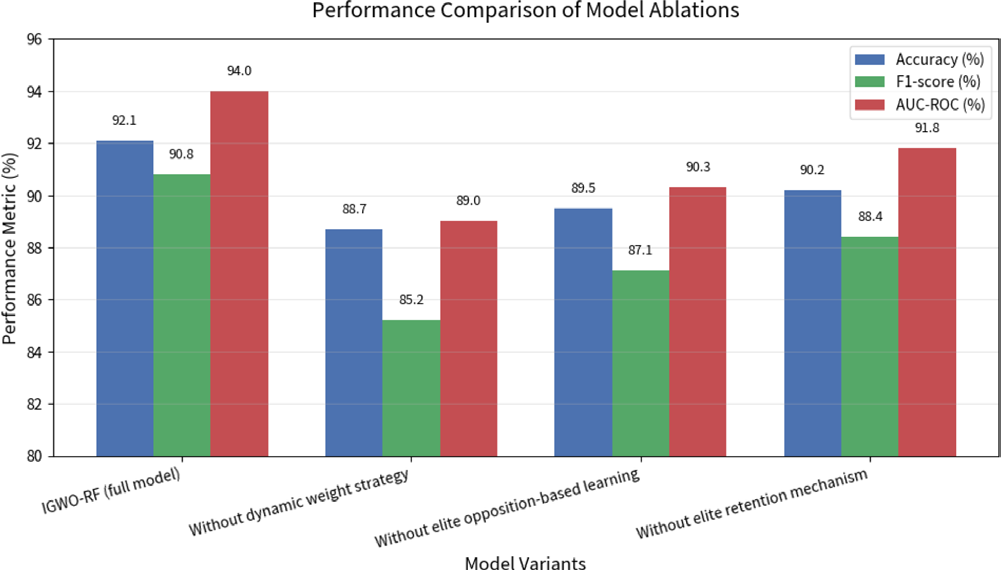
The performance comparison of model ablations.

**Figure 5.**
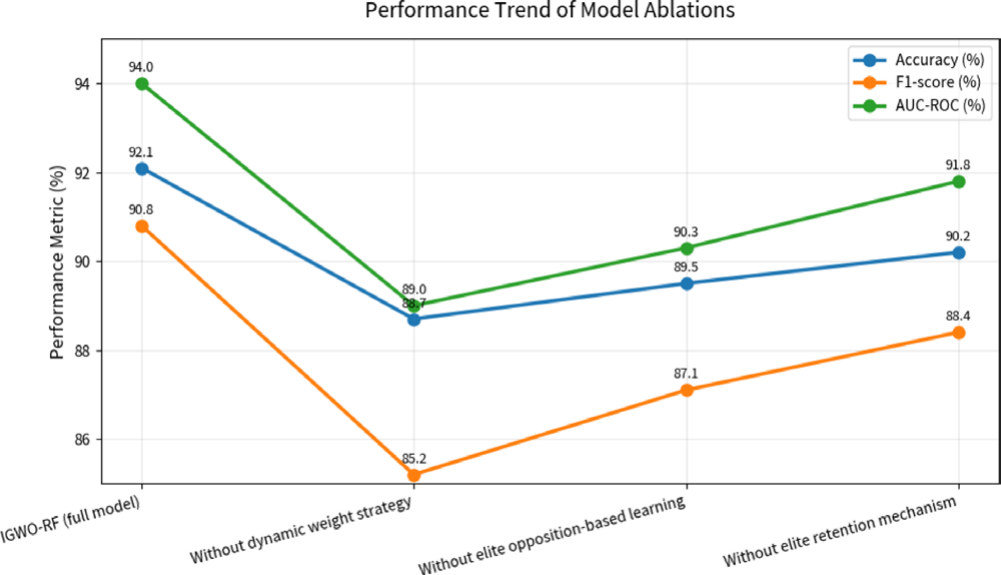
The performance trend of model ablations.

Figure 4 shows the performance comparison of model ablations. Figure 5 shows the performance trend of model ablations. The results indicate that all 3 improvements contribute to the models performance, with the dynamic weight strategy having the most significant impact (2.5% accuracy reduction when removed). This confirms that each component plays a crucial role in enhancing the optimization capability of the algorithm.

### 4.6. Discussion of results

The experimental results demonstrate that the proposed IGWO–RF model outperforms traditional machine learning methods and other hybrid models in children’s health prediction. Its significant improvement over standard RF highlights the effectiveness of IGWO-based hyperparameter optimization in addressing manual tuning limitations and enhancing model generalization. Compared with PSO-RF, the superior performance of IGWO–RF stems from the dynamic balance between exploration and exploitation achieved by the IGWO algorithm, along with its faster convergence speed, which reduces computational complexity and enhances suitability for clinical applications with large datasets. However, it is important to acknowledge that the dataset was sourced from a single region, which may limit the generalizability of our findings to broader populations. Future studies should validate the model on multi-center datasets from diverse geographic and demographic backgrounds to confirm its robustness and applicability.

The feature importance analysis provides valuable clinical insights into children’s health determinants. The dominant role of BMI aligns with current public health concerns about childhood obesity, while the significant impact of environmental factors (PM2.5, green coverage) emphasizes the importance of addressing environmental health disparities. The identified interaction between BMI and exercise time suggests that interventions targeting both factors simultaneously may be more effective than addressing them individually.

Furthermore, the SHAP interpretation framework offers a promising pathway for integrating the IGWO–RF model into pediatric decision support systems. By quantifying feature contributions and interactions, SHAP values can enhance transparency and trust in model predictions, which is critical for clinical adoption. For example, SHAP-based visualizations, such as summary plots and dependence plots, could be embedded into electronic health record systems to provide clinicians with intuitive explanations of key risk factors for individual patients. Such integration could enable personalized risk stratification and proactive interventions. For instance, if a patient’s SHAP analysis indicates high PM2.5 exposure contribution, clinicians might recommend environmental modifications alongside lifestyle counseling. However, practical implementation requires addressing challenges such as system interoperability, data privacy, and clinician training. Future work should focus on developing user-friendly interfaces and validating SHAP’s utility in real-world clinical workflows to ensure seamless integration.

The ablation study confirms the rationality of the proposed improvements to the GWO algorithm, with each component contributing to the overall performance gain. This validates the design choices in developing the IGWO algorithm for hyperparameter optimization in healthcare prediction tasks.

## 5. Conclusion

In this study, an IGWO–RF model was proposed for children’s health prediction. The model integrated the strengths of the improved GWO algorithm for hyperparameter optimization and the RF algorithm for prediction. Experimental results demonstrated that the IGWO–RF model achieved high predictive performance for children’s health classification in this dataset, with an accuracy of 92.1% and a F1-score of 90.8% on the test set, significantly outperforming the standard RF model and the PSO-RF model. This highlights the model’s capability in handling complex pediatric health data, though its applicability is contingent on further validation.

The feature importance analysis using SHAP values provided valuable insights into the key determinants of children’s health. BMI, daily exercise time, and PM2.5 exposures were identified as the top 3 influential factors, highlighting the significance of lifestyle and environmental factors in children’s health. The interaction analysis further revealed the complex relationships between these factors, emphasizing the need for comprehensive interventions. The ablation study validated the effectiveness of each improvement in the IGWO algorithm. The dynamic weight strategy, elite opposition-based learning, and elite retention mechanism all contributed to the enhanced performance of the model, demonstrating the rationality of the proposed algorithmic improvements.

However, this study has several limitations. The data was sourced from a single region, which may limit the generalizability of the model to other populations. Additionally, the cross-sectional nature of the dataset restricts the ability to establish causal relationships between variables. The IGWO–RF model is potentially useful for children’s health risk stratification, pending external validation, with excellent predictive performance, and its methodological framework could be extended to early warning systems for other chronic diseases upon further validation. Future research directions should focus on incorporating longitudinal data from multiple regions to better capture the dynamic changes in children’s health over time. External validation and longitudinal data integration would further confirm its clinical applicability. Moreover, integrating deep-learning techniques could potentially improve the model’s performance in handling complex data patterns.

## Author contributions

**Conceptualization:** Huan Xu.

**Formal analysis:** Huan Xu.

**Investigation:** Huan Xu.

**Methodology:** Huan Xu.

**Project administration:** Junying Hu.

**Writing – original draft:** Huan Xu.

**Writing – review & editing:** Huan Xu, Junying Hu.

## References

[1] World Health Organization. Global health observatory data repository. https://www.who.int/data/gho. Accessed 2024.

[2] Doctor J MacEwan JP. Limitations of traditional health technology assessment methods and implications for the evaluation of novel therapies. Curr Med Res Opin. 2017;33:1635–42. 28756684 10.1080/03007995.2017.1359151

[3] Ganatra HA. Machine learning in pediatric healthcare: current trends, challenges, and future directions. J Clin Med. 2025;14:807. 39941476 10.3390/jcm14030807 PMC11818243

[4] Senior M Fanshawe T Fazel M Fazel S. Prediction models for child and adolescent mental health: a systematic review of methodology and reporting in recent research. JCPP Adv. 2021;1:e12034. 37431439 10.1002/jcv2.12034 PMC10242964

[5] Colmenarejo G. Machine learning models to predict childhood and adolescent obesity: a review. Nutrients. 2020;12:2466. 32824342 10.3390/nu12082466 PMC7469049

[6] Bhardwaj P Tyagi A Tyagi S Antão J Deng Q. Machine learning model for classification of predominantly allergic and non-allergic asthma among preschool children with asthma hospitalization. J Asthma. 2023;60:487–95. 35344453 10.1080/02770903.2022.2059763

[7] El-Bashbishy AES El-Bakry HM. Pediatric diabetes prediction using deep learning. Sci Rep. 2024;14:4206. 38378741 10.1038/s41598-024-51438-4 PMC11291908

[8] Azeta A Edward C Ekpo R. Interactive machine learning framework for predicting asthma health conditions using XGBoost[C]. 2024 international conference on emerging trends in networks and computer communications (ETNCC). IEEE. 2024:849–56.

[9] Lundberg S Lee S. A unified approach to interpreting model predictions. Nat Mach Intell. 2019;1:252–60.

[10] Buxton EK Vohra S Guo Y Fogleman A Patel R. Pediatric population health analysis of southern and central Illinois region: a cross sectional retrospective study using association rule mining and multiple logistic regression. Comput Methods Programs Biomed. 2019;178:145–53. 31416543 10.1016/j.cmpb.2019.06.020

[11] Triantafyllidis A Polychronidou E Alexiadis A. Computerized decision support and machine learning applications for the prevention and treatment of childhood obesity: a systematic review of the literature. Artif Intell Med. 2020;104:101844. 32498995 10.1016/j.artmed.2020.101844

[12] Wang J Yang Y Gong X. Interpretable machine learning for allergic rhinitis prediction among preschool children in Urumqi, China. Sci Rep. 2024;14:22281. 39333659 10.1038/s41598-024-73733-w PMC11437280

[13] Zames G. Genetic algorithms in search, optimization and machine learning. Inf Tech J. 1981;3:301.

[14] Kennedy J Eberhart R. Particle swarm optimization. Proceedings of ICNN’95-international conference on neural networks. ieee, 1995;4:1942–1948.

[15] Mirjalili S Mirjalili SM Lewis A. Grey wolf optimizer. Adv Eng Softw. 2014;69:46–61.

[16] Teng Z Lv J Guo L. An improved hybrid grey wolf optimization algorithm. Soft Comput. 2019;23:6617–31.

[17] Li L Qiao J Yu G. Interpretable tree-based ensemble model for predicting beach water quality. Water Res. 2022;211:118078. 35066260 10.1016/j.watres.2022.118078

[18] Xia T Han K. Machine learning prediction model with shap interpretation for chronic bronchitis risk assessment based on heavy metal exposure: a nationally representative study. BMC Pulm Med. 2025;25:252. 40405146 10.1186/s12890-025-03724-8 PMC12096596

